# Erectile Dysfunction in Chronic Prostatitis/Chronic Pelvic Pain Syndrome: Outcomes from a Multi-Center Study and Risk Factor Analysis in a Single Center

**DOI:** 10.1371/journal.pone.0153054

**Published:** 2016-04-27

**Authors:** Yadong Zhang, Tao Zheng, Xiang'an Tu, Xin Chen, Zhu Wang, Shengfu Chen, Qiyun Yang, Zi Wan, Dayu Han, Haipeng Xiao, Xiangzhou Sun, Chunhua Deng

**Affiliations:** 1 Department of Urology, 1^st^ Affiliated Hospital of Sun Yat-sen University, Guangzhou, 510080, Guangdong Province, China; 2 Department of Ultrasound Medicine, Endocrinology, 1^st^ Affiliated Hospital of Sun Yat-sen University, Guangzhou, 510080, Guangdong Province, China; 3 Department of Endocrinology, 1^st^ Affiliated Hospital of Sun Yat-sen University, Guangzhou, 510080, Guangdong Province, China; Northwestern University, UNITED STATES

## Abstract

The aim of this study was to investigate the prevalence of erectile dysfunction (ED) in patients with chronic prostatitis/chronic pelvic pain syndrome (CP/CPPS) and explore the influence of UPOINT domains, National Institutes of Health-CP symptom index (NIH-CPSI) and other factors on ED prevalence. This was a prospective study of consecutive patients with CP/CPPS seen at 11 tertiary hospitals during January–July 2014. ED was diagnosed as a score of<21 on the International Index of Erectile Function (IIEF-5). Patients from one center were evaluated by the UPOINT system and NIH-CPSI. Each patient was assessed using clinical examination, asocio-demographic questionnaire, the Patient Health Questionnaire (PHQ), the Pain Catastrophizing Scale (PCS), NIH-CPSI and IIEF-5.1406 patients from 11 centers (mean age, 32.18 years; range 18–60 years) were enrolled. ED was found in 638/1406 patients (45.4%), and was categorized as mild in 291(45.6%), moderate in 297(46.6%) and severe in50(7.7%). 192 patients from one center(mean age,31.3 years; range 18–57 years) were further studied.IIEF-5 score correlated negatively with NIH-CPSI(*r* = 0.251), PHQ (*r* = 0.355) and PCS (*r* = 0.322)scores (P<0.001).PHQ score correlated positively with NIH-CPSI (*r* = 0.586) and PCS(*r* = 0.662) scores (P<0.001).NIH-CPSI, PHQ, PCS and IIEF-5 scores did not differ significantly between class IIIA and IIIB CP/CPPS. Multivariate logistic regression showed that UPOINT psychological (P) domain and NIH-CPSI symptom severity were independent risk factors for ED in CP/CPPS. It is concluded that psychological factors and symptom severity are independent risk factors for ED in CP/CPPS.

## Introduction

Chronic prostatitis/chronic pelvic pain syndrome (CP/CPPS) is a common urological disease that has significant economic costs and a severe impact on patient quality of life[1, 2]. The prevalence of CP/CPPS has been estimated to be between 2.2% and 13.8%[3]. Several studies have reported a high prevalence of erectile dysfunction(ED) in patients with CP/CPPS. For example, the prevalence of ED was reported as 31.5% in764 patients with CP/CPPS in Italy[3], 48.3%in 296 patients with CP/CPPS in Malaysia[4], and 35.1% in a multi-center study of patients with CP in China[5]. From the other perspective, the prevalence of CP/CPPS in patients with ED is also higher. For example, a case-control study in Taiwan found that 8.6% of patients with ED had a history of CP/CPPS[6], compared with 2.5% of patients without ED. Regression analysis showed that a previous diagnosis of CP/CPPS was 3.62-fold more likely in patients with ED than in those without ED. The aforementioned research indicates that ED is closely related to CP/CPPS, but there have been few studies exploring the factors that predict the occurrence of ED in patients with CP/CPPS[7].

CP/CPPS is a heterogeneous condition with a variety of symptoms and, potentially, a variety of etiologies[8]. No validated predictors or biomarkers are currently available that can help classify patients with CP/CPPS and subsequently direct appropriate therapy. The National Institutes of Health-Chronic Prostatitis Symptom Index (NIH-CPSI)is a 9-question validated questionnaire that allows quantification of pain, voiding symptoms and quality of life, and is the most commonly used system for evaluating the symptoms of chronic prostatitis[9]. The NIH-CPSI has some limitations in that it does not assess sexual function, the presence of infection, or the presence of social or psychological abnormalities, even though symptoms relating to these are very common in patients with CP/CPPS. Based on the limitations of the NIH-CPSI, the complexity of CP/CPPS etiology and the limited effect of monotherapy, Shoskes et al.[10] developed a 6-point clinical phenotyping system called UPOINT which contains the following clinical domains: urinary symptoms (U), psychosocial dysfunction (P), organ-specific (O), infection (I), neurological/systemic (N) and tenderness of muscles (T). Each domain has been clinically defined, linked to specific mechanisms of symptom production or progression, and associated with specific therapy[11–13]. However, these phenotypes are qualitative, with each domain scored as ‘yes’ or ‘no’; thus, UPOINT does not consider the intensity of these symptoms or their interference with function, and hence lacks important information[14]. The combined use of NIH-CPSI and UPOINT can more fully assess the clinical characteristics of CP/CPPS.

The factors predicting the occurrence of ED in patients with CP/CPPS remain unclear. In particular, the associations of UPOINT domains, NIH-CPSI and various other factors with the occurrence of ED in patients with CP/CPPS have yet to be determined. The purpose of the present study was to investigate the prevalence of ED in patients with CP/CPPS, and explore the influence of UPOINT domains, NIH-CPSI and other factors on the occurrence of ED in patients with CP/CPPS.

## Materials and Methods

### Patients and study design

**Fig 1:**
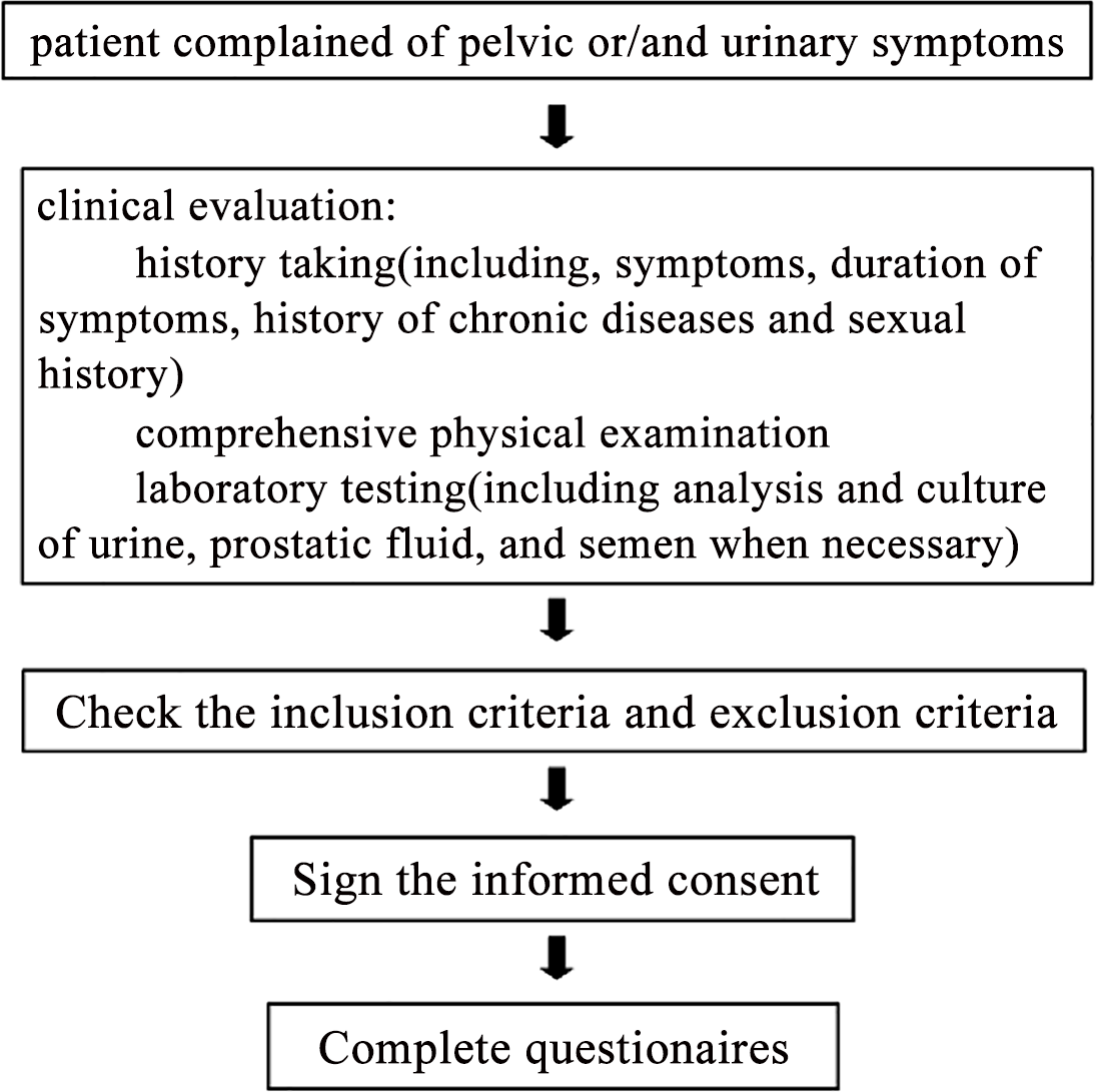
Study flowchart for patients enrollment.

This was a prospective study of consecutive with CP/CPPS attending urology or andrology clinics at 11 tertiary referral hospitals between January 2014 and July 2014. The inclusion criteria were: 1) a patient diagnosis of CP/CPPS, based on the following diagnostic criteria[15]: long-term and recurrent pelvic pain or discomfort for longer than 3 months as the main manifestation, with diseases affecting urination excluded (such as the presence of active urethritis, urogenital cancer, urinary tract disease, functionally significant urethral stricture, or neurological disease affecting the bladder); 2) age ≥18 years; and 3) in a stable sexual partnership. CP/CPPS was categorized into one of two types, subtype IIIA (inflammatory) and IIIB (non-inflammatory), based on the prostatic fluid leukocyte count[15]. The exclusion criteria were:1) diseases influencing sexual function, including psychiatric disorders, hypertension, diabetes mellitus, coronary artery disease, spinal cord injury, peripheral vascular diseases and endocrine disease; and 2)use of drugs affecting sexual function, including PDE5 inhibitors (sildenafil, vardenafil or tadalafil), hormone drugs (testosterone undecanoate or estradiol) and antidepressants (fluoxetine, paroxetine or sertraline). Local ethical approval was obtained from the Ethics Committee of the First Hospital affiliated to Sun Yat-Sen University (approval ID: [2014]24). All patients were informed about the study and gave their informed consent before inclusion in the study (Fig 1).

#### Data collected from all patients at the 11 tertiary hospitals

All patients with CP/CPPS from the 11 centers received clinical evaluation involving history taking (including symptoms, duration of symptoms, therapeutic interventions, history of chronic diseases and sexual history), comprehensive physical examination (of the lower abdomen, rectum, prostate, genitalia, and neurological system when necessary), and laboratory testing (including analysis and culture of urine, prostatic fluid, and semen when necessary). All patients were also asked to complete questionnaires in order to obtain the following details: 1) patient general information, including age, height and weight; 2) NIH-CPSI score, which evaluates the severity of CP/CPPS symptoms(mild, 5–14 points;moderate,15–29 points; and severe,30–43 points)[12]; and 3) International Index of Erectile Function 5(IIEF-5)score, which evaluates erectile function (diagnosis of ED,<22 points; mild ED,12–21 points; moderate ED, 8–11points; and severeED,5–7 points)[12].

#### Additional data collected only from patients at the First Hospital affiliated to Sun Yat-Sen University

In addition to receiving the clinical evaluations and questionnaires described above, patients enrolled a tone of the 11 centers (the First Hospital affiliated to Sun Yat-Sen University) were also asked to complete the following questionnaires: 1) patient health questionnaire (PHQ); and 2) pain catastrophizing scale (PCS)questionnaire. The PHQ is used as a screening instrument to diagnose common mental health disorders in a primary care setting; scores range from 0 to 27, and scores of 5, 10, 15 and 20 represent the (inclusive) cutoff points for minor, moderate, major and severe depression, respectively[16]. The PCS assesses three measures of negative thoughts associated with pain: rumination or worry (for instance, excessive attention to pain sensations), magnification(for example, reinforcing the threats of pain sensations), and helplessness (for instance, awareness of the inability to cope with the symptoms of pain); a total score of 38 points or more is taken to represent a clinically relevant level of catastrophizing[17].

According to the clinical data and questionnaires, a yes/no classification for each of the six UPOINT domains was made for each individual patient. In brief, urinary symptoms (U) were considered positive if the patient had a NIH-CPSI urinary score>4 or complained of bothersome urgency, frequency or nocturia; psychosocial dysfunction (P) was considered positive if the PHQ score was <5 or the PCS was>37;organ-specific findings (O) were considered positive if the patient had tenderness localized to the prostate, leukocytosis in the prostatic fluid, hematospermia, or extensive prostatic calcifications; the infection domain (I) was considered positive if gram-negative bacilli or *Enterococcus* was found in expressed prostatic fluid orpost-massage urine in the absence of urinary tract infection; the neurological/systemic domain (N) was considered positive if pain was experienced outside the abdomen and pelvis, or concurrent diagnoses of fibromyalgia, chronic fatigue syndrome or irritable bowel syndrome were made; and the tenderness domain (T) was considered positive if palpable muscle spasm or trigger points were present in the abdomen or pelvic floor[10].

All the scales described above are the most widely used systems for evaluating the symptoms, mental disorders, and erectile functions of patients with CP/CPPS, and their adapted Chinese versions have been verified to have excellent reliability and validity. Researchers in the present study were all specialists from departments of urology orandrology, and had received standardized training with regard to their role in the study. The respondents were fully informed of the contents and significance of the surveys and voluntarily completed the questionnaires within 15–20 minutes. For respondents with a low education level, investigators described and explained the contents of the questionnaires item by item, and then recorded the results according to the oral responses of the respondent. The survey results were inputted and checked independently by two investigators to guarantee the accuracy of the data.

### Statistics analysis

All statistical comparisons were made using SPSS 19.0 (IBM Corp., Armonk, NY, USA). Comparisons between two groups were made using the Student’s t-test. Correlation between two variables was assessed by Pearson correlation analysis. Logistic regression analysis, with ED as the dependent variable and age, BMI, symptom duration, symptom severity (NIH-CPSI score) and UPOINT domains as independent variables, was used to analyze the associations between these factors and the occurrence of ED in patients with CP/CPPS.P < 0.05 was considered significant.

## Results

### The prevalence of ED in patients with CP/CPPS: multi-center results

A total of 1406 patients with CP/CPPS, with a mean age of 32.18 years (range, 18–60 years), were enrolled at the 11 centers. A total of 638 of these 1406 patients (45.4%) had ED, which was mild in 291(45.6%), moderate in 297(46.6%) and severe in 50(7.7%).

### Demographic and clinical characteristics of192 patients from a single center

The demographic and clinical characteristics (including PHQ, PCS, NIH-CPSI and IIEF-5 scores) of 192 patients enrolled at the First Affiliated Hospital of Sun Yat-Sen University are presented in Table 1 (comparisons between patients with ED and those without ED are also shown). These192 patients had a mean age of 31.3 years (range, 18–57 years), a mean BMI of 22.78±3.16 kg/m^2^, and a median symptom duration of 20.5 months(range,3–165 months). A total of 91 of the 192 patients (47.4%) had ED. There were no significant differences between this subgroup of 192 patients and the full cohort of 1406 patients in age, BMI, disease course and prevalence of ED.

**Table 1:** Demographic and clinical characteristics of 192 patients enrolled ata single center.

Characteristic	ED group N = 91	Non-ED group N = 101	TotalN = 192	P value
Age, years	31.4±7.6	31.2±6.9	31.3±7.2	0.968
BMI, kg/m^2^	22.4±2.9	23.1±3.3	22.8±3.2	0.093
Duration of symptoms, months	21.2±28.7	19.9±25.3	20.5±26.9	0.968
*CP/CPPS diagnosis*				
Inflammatory (Type IIIA)	48(52.7%)	54(53.5%)	102(53%)	0.921
Non-inflammatory (Type IIIB)	43(47.3%)	47(46.5%)	90(47%)	
PHQ	9.4±6.3	5.2±4.7	7.2±5.9	<0.001
PCS	14.6±12.1	7.9±8.1	11.1±10.7	<0.001
NIH-CPSI	22.2±7.1	18.8±6.2	20.5±6.9	<0.001
*Symptom severity*				
Mild	12(13.2%)	29(28.7%)	41 (21.4%)	0.001
Moderate	64(70.3%)	67(66.3%)	131 (68.2%)	
Severe	15(16.5%)	5(5%)	20 (10.4%)	
IIEF-5	16.5±4.2	23.1±1.2	20.0±4.5	<0.001
*Positive UPOINT domains*				
Urinary(U)	66(72.5%)	70(69.3%)	136 (70.8%)	0.624
Psychosocial(P)	69(75.8%)	46(45.5%)	115 (59.9%)	<0.001
Organ specific(O)	70(76.9%)	85(84.2%)	155 (80.7%)	0.204
Infection(I)	24(26.4%)	21(20.8%)	45 (23.4%)	0.362
Neurological/systemic(N)	48(52.7%)	39(38.6%)	87 (45.3%)	0.049
Tenderness of muscles(T)	60(65.9%)	60(59.4%)	120 (62.5%)	0.351

Data presented as mean ± standard deviation or n (%). Abbreviations: BMI, body mass index;CP/CPPS, chronic prostatitis/chronic pelvic pain syndrome; ED, erectile dysfunction; IIEF, International Index of Erectile Function; NIH-CPSI, National Health Institutes Chronic Prostatitis Symptom Index; PCS, Pain Catastrophizing Scale; PHQ, Patient Health Questionnaire.

The subgroup of 192 patients had a mean PHQ score of 7.2±5.9, a mean PCS score of11.1±10.7, a meanIIEF-5 score of20.0±4.5, and a mean NIH-CPSI score of 20.5±6.9 (Table 1). The severity of the CP/CPPS, evaluated from the NIH-CPSI score, was mild in 41 patients (21.4%), moderate in 131(68.2%), and severe in 20(10.4%)(Table 1). The positive rate for each UPOINT domain was as follows: U, 70.8%; P, 59.9%; O,80.7%; I, 23.4%;N, 45.3%; and T, 62.5% (Table 1).

Among the subgroup of 192 patients, there were no significant differences in age, BMI or duration of symptoms between those with ED and those without ED (Table 1). Patients with ED had significantly higher PHQ, PCS, NIH-CPSI and IIEF-5 scores, and higher rates of positivity in the P and N domains of UPOINT, than those without ED (Table 1).

Patients with ED and those without ED did not differ significantly with regard to the proportion of patients with type IIIA and type IIIB CP/CPPS (Table 1). In addition, there were no significant differences in PHQ, PCS, NIH-CPSI and IIEF-5 scores between patients with type IIIA CP/CPPS and those with type IIIB disease (Table 2).

**Table 2:** Comparison of NIH-CPSI, PHQ, PCS and IIEF-5 scores between patients with type IIIA and IIIB CP/CPPS.

Index	IIIA patients (n = 102)	IIIB patients (n = 90)	Pvalue
NIH-CPSI	20.75±7.550	20.11±6.018	0.518
PHQ	7.92±6.442	6.38±5.085	0.065
PCS	12.23±12.009	9.80±9.006	0.113
IIEF-5	19.89±4.732	19.97±4.156	0.908

Abbreviations:CP/CPPS, chronic prostatitis/chronic pelvic pain syndrome; IIEF, International Index of Erectile Function; NIH-CPSI, National Health Institutes Chronic Prostatitis Symptom Index; PCS, Pain Catastrophizing Scale; PHQ, Patient Health Questionnaire.

### Correlation analyses

Pearson correlation analysis revealed that the IIEF-5 score showed negative correlations with the NIH-CPSI score (Pearson correlation coefficient, *r* = 0.251), PHQ score (*r* = 0.355) and PCS score (*r* = 0.322) (P <0.001), but no significant correlations with age and duration of symptoms. The PHQ score correlated positively with the NIH-CPSI score (*r* = 0.586) and PCS score (*r* = 0.662) (P < 0.001), but showed no significant correlation with age and duration of symptoms.

#### Factors associated with the presence of ED in patients with CP/CPPS

Univariate logistic regression analysis with ED as the dependent variable and age, BMI, symptom duration, symptom severity (NIH-CPSI score) and UPOINT domains as independent variables showed that UPOINT psychological(P) domain, Neurological/systemic(N) and symptom severity were risk factors for ED in patients with CP/CPPS (Table 1).

Multivariate logistic regression analysis showed that the UPOINT psychological(P) domain and symptom severity were independent risk factors for ED in patients with CP/CPPS (Table 3).

**Table 3:** Multivariate logistic regression analysis of the influence of UPOINT domains and other factors on ED prevalence in men with CP/CPPS.

Variable	Odds ratio (95% CI)	P value
Age	1.014 (0.967–1.063)	0.559
BMI	0.919 (0.827–1.023)	0.121
Symptom duration	1.000 (0.988–1.013)	0.946
Severity of symptoms	2.599 (1.341–5.038)	0.005
Urinary(U)	0.730 (0.345–1.543)	0.410
Psychosocial(P)	3.804 (1.898–7.622)	0.000
Organ specific(O)	0.473 (0.207–1.082)	0.076
Infection(I)	1.731 (0.812–3.690)	0.156
Neurological/systemic(N)	1.107 (0.561–2.184)	0.770
Tenderness of muscles(T)	0.931 (0.468–1.853)	0.839

Abbreviations: BMI, body mass index; CI, confidence interval.

## Discussion

The main findings of the present study were that nearly half the patients with CP/CPPS had ED, and that just over half of these had moderate or severe ED. Furthermore, patients with ED had higher PHQ, PCS, NIH-CPSI and IIEF-5 scores than those without ED, as well as higher rates of positivity in the P and N domains of UPOINT. In addition, UPOINT P domain and symptom severity were independent risk factors for ED in patients with CP/CPPS. These novel data provide further insights into the factors predicting ED in patients with CP/CPPS.

ED, defined as the inability to obtain or maintain an erection sufficient for adequate sexual performance, is a highly prevalent complaint among patients with CP/CPPS[18]. Previous studies have reported a prevalence of ED in patients with CP/CPPS of 31.5–48.3%[3–5]. The present multi-center study determined that the prevalence of ED in patients with CP/CPPS was 45.4%, which is similar to previous findings. In the general population, the prevalence of ED ranges from 1–10% in those below the age of 40 years, and from 2–9% in those aged 40–49 years (although values as high as 15% have been reported). In the 50–59 years age group, the reported prevalence varies widely from rates as low as 6–18% to rates as high as 32–35%[19]. Nonetheless, the prevalence of ED in patients with CP/CPPS is higher than that in the general population, indicating that CP/CPPS has a negative influence on erectile function. It is noteworthy that, in the present study, ED in patients with CP/CPPS was mostly at the mild and moderate level.

In the present study, multivariate logistic regression the psychological (P) domain of UPOINT was an independent risk factor for ED in patients with CP/CPPS. Many previous studies have found that CP/CPPS is commonly accompanied by depression, anxiety, hypochondriasis, relationship disorders and even suicidal tendency, and that patients with these psychological manifestations have a high prevalence of ED[20–22]. However, these previous studies did not explore the relationship between ED and psychological disorders in patients with CP/CPPS. Results from the Massachusetts Male Aging Study (MMAS) confirmed that the relationship between depressive symptoms and ED in middle-aged men is robust and independent of important aging and para-aging confounders, such as demographic, anthropometric and lifestyle factors, health status, medication use and hormones[23]. Based on these studies, it could be hypothesized that ED is related to psychological disorders in patients with CP/CPPS. The present study provides strong evidence for this hypothesis.

Multivariate logistic regression demonstrated that symptom severity (evaluated by NIH-CPSI) was also an independent risk factor for ED in patients with CP/CPPS. This finding is consistent with the research of Marszalek et al.[24], who reported that an NIH-CPSI score in the upper quartile was associated with 8.3-fold increased odds of ED. However, the study of Marszalek et al. did not take into account possible interference from confounding factors such as psychological disorders, which are known to be very common in patients with CP/CPPS and to negatively affect erectile function. The present study comprehensively evaluated the clinical characteristics of patients with CP/CPPS using the NIH-CPSI and UPOINT systems, and explored the influence of these clinical characteristics and demographic factors on ED in these patients. By taking into account possible interference from confounding factors, the results of the present study may be considered to be more reliable than those of previous investigations.

The IIEF-5 score showed a negative correlation with NIH-CPSI, PHQ and PCS scores, indicating that the severity of ED correlates with the severity of CP/CPPS symptoms and psychological disorders. Aubin et al.[25] analyzed factors related to sexual function in patients with CPPS, including pain status and psychological adaptation, and showed that erectile function decreased with increasing pain symptoms and stress appraisal. Marszalek et al.[24]also found that the IIEF-5score decreased with an increase in overall NIH-CPSI score, as well as with an increase in the pain, urinary symptoms and quality of life subdomains of NIH-CPSI. These findings are consistent with ours, which demonstrated that the severity of CP/CPPS symptoms and psychological disorders had a negative influence on erectile function.

Although it remains difficult to establish causality between CP/CPPS symptoms and psychological disorders, various studies have identified a correlation between these. A study in South Korea[26] suggested that the depression score increased with urinary symptoms and pain symptoms. McNaughton et al.[2] found that patients with CP/CPPS who had more severe symptoms were more likely to have lower mental component summary scores on the 12-item Short Form. The present study also found that the PHQ score correlated positively with the NIH-CPSI score. Thus, it is possible that severe CP/CPPS symptoms might lead to ED through psychological factors.

In the present study, multivariate logistic regression showed that the urinary(U) domain of UPOINT was not a risk factor for ED. This finding is not consistent with previous studies reporting that lower urinary tract symptoms (LUTS) were independent risk factors for ED[27–30]. Possible explanations for this inconsistency are as follows. First, there may be differences between studies in the subjects enrolled: we chose younger populations (18–57 years, mean age 31.30±7.20 years) where as middle-aged and older patients were recruited in the studies of LUTS. Second, LUTS and ED may have common pathological bases, which include the nitric oxide synthase/nitric oxide (NOS/NO) theory, autonomic nervous system hyperfunction and metabolic syndrome hypothesis, and pelvic arteriosclerosis theory. These common pathological factors correlate mainly with aging and geriatric conditions that are accompanied by LUTS[29, 30]. It is possible that these pathological factors do not exist in younger patients with CP/CPPS. Of course, more research is needed for further clarification.

We also found that the organ-specific (O) domain of UPOINT was not a risk factor for ED, and that there was no significant difference in the IIEF-5 score between type IIIA and type IIIB patients. These results indicate that local inflammation of the prostate has no effect on ED in patients with CP/CPPS. This study also found no differences in NIH-CPSI, PHQ and PCS scores between type IIIA and type IIIB patients, suggesting that local inflammation of the prostate has no influence on CP/CPPS symptoms and psychological disorders. These observations can be used to explain why local inflammation of the prostate has no effect on ED.

The present study has some limitations. First, this was a cross-sectional study that only investigated the prevalence of ED in CP/CPPS, and was not able to determine causality. Second, the analysis of risk factors for ED was undertaken only in 192 patients from one center; thus, our findings may not be generalizable to the general population in China or other countries. Third, since some individuals may be reluctant to visit a physician for CP/CPPS or sexual problems, the inclusion of patients in the present study may have been biased towards those with more bothersome symptoms; hence, selection bias cannot be ruled out. Fourth, it cannot be excluded that other covariates not adjusted for in our logistic regression analysis may have confounded our results. Large-scale, longitudinal and multi-center investigations are merited to confirm and extend our findings.

In conclusion, patients with CP/CPPS had a higher rate of ED than that reported for the general population; in most cases, ED was mild or moderate in severity. Psychological factors and symptom severity were independent risk factors for ED in patients with CP/CPPS. The degree of ED correlated with the severity of the psychological disorder and CP/CPPS symptoms. Local inflammation of the prostate had no effect on erectile function in patients with CP/CPPS. Thus, it is important that psychological status and erectile function are evaluated in patients with CP/CPPS, particularly those with severe symptoms. In addition, these patients should be encouraged to accept early symptomatic treatment or psychological counseling.
